# Hypothalamo−Pituitary Insufficiency Associated with Ectrodactyly−Ectodermal Dysplasia−Clefting Syndrome

**DOI:** 10.4274/jcrpe.v1i5.252

**Published:** 2009-08-08

**Authors:** Nihal Hatipoğlu, Selim Kurtoğlu, Derya Büyükayhan, Mustafa Akçakuş

**Affiliations:** 1 Department of Pediatrics, Pediatric Endocrinology Unit, Erciyes University Medical Faculty, Kayseri, Turkey; 2 Department of Pediatrics, Neonatology Unit, Cumhuriyet University Medical Faculty, Sivas, Turkey; +90 352 438 00 76nihalhatipoglu@yahoo.comDeartment of Pediatrics, Pediatrc Endocrinology Unit Erciyes University Medical Faculty, Kayseri, Turkey

**Keywords:** EEC syndrome, hypothalamo−pituitary insufficiency

## Abstract

Ectrodactyly−ectodermal dysplasia−clefting (EEC) syndrome is characterized by ectodermal dysplasia, ectrodactyly and facial clefting with multiple congenital anomalies such as urinary tract anomaly, lacrimal duct obstruction, and hearing loss. This syndrome is a rare disease transmitted by autosomal dominant inheritance with variable penetrance. Clinical expression is variable. In EEC syndrome with midline defect hypothalamo−pituitary endocrinopathy is expected, however hormonal disorders in EEC syndrome have rarely been reported. We present two patients with EEC syndrome associated with hypothalamo−pituitary insufficiency.

**Conflict of interest:**None declared.

## INTRODUCTION

Ectrodactyly−ectodermal dysplasia−clefting (EEC) syndrome is a rare autosomal dominant genetic syndrome. The EEC syndrome was first described in 1936 by Cockayne who reported a case with dacrocystitis and orofacial clefting (1). Rudiger et al (2) used the acronym and described a girl with trimelic ectrodactyly, ectodermal features involving hair, teeth, and nails, and bilateral cleft lip and palate. 

The syndrome is usually inherited as an autosomal dominant trait, although it may occur sporadically (3). 

The main clinical features of this syndrome are ectrodactyly (lobster−claw deformity), ectodermal dysplasia and cleft lip. In addition to these cardinal features, lacrimal duct abnormalities, urogenital anomalies, mental retardation and conductive deafness may coexist in varying degrees (4, 5). 

We present in this paper multiple pituitary hormone deficiency in two patients with EEC syndrome.

## PATIENT 1

**Figure 1:**
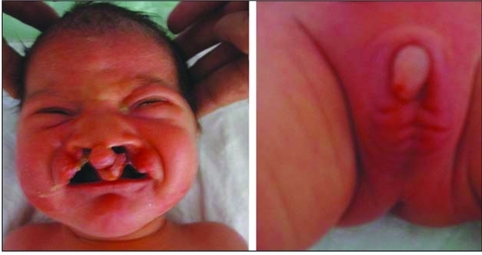
Bilateral cleft lip and plate, hypertelorism, micropenis and cryptorchidism of case 1

**Figure 2:**
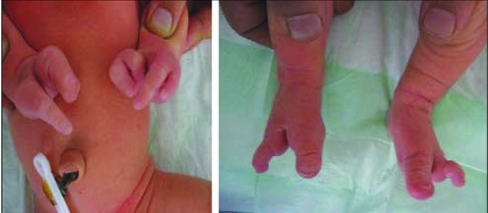
Ectrodactyly deformity of hands and feet of case 1

A male infant was born to healthy, unrelated parents at 40 weeks of gestation. His father was 31 years and the mother was 29 years old. He was born by vaginal delivery and cried spontaneously. The mother had no significant antenatal history. The other offsprings were normal. There were no similar complaints in the other members of the family. The baby was admitted to the neonatal care unit with the diagnosis of dysmorphic baby. Physical examination revealed the following: head circumference 35 cm (50^th^ centile), chest circumference 32 cm (25−50^th^ centile), weight 3300 gr (50^th^ centile), and length 49 cm (50−75^th^ centile). His skin was dry and thin. He had low−set ears, midline bilateral complete cleft lip and palate and hypertelorism. His penile length was 1.6 cm and had bilateral cryptorchidism (Figure 1). He had lobster claw deformity and ectrodactyly of the hands and feet. His both hands were split between the third and fourth fingers and the left hand had 4 fingers; the first and second fingers were bilaterally syndactylous (Figure 2). The hair and nails were normal. The lacrimal ducts were intact. No other systems were clinically involved. His echocardiography and renal ultrasonography were evaluated as normal. His cranial magnetic resonance imaging showed agenesis of the septum pellucidum and adenohypophyseal height was 2.5 mm (normal range 3.5±0.5 mm) (6). His hormonal levels including FSH: 0.00 UI/l (2.91 ± 4.38, median 1.5 UI/l), LH: 0.00 UI/l (2.31±2.29, median 1.4 UI/l), total testosterone: 24 ng/dl (75−400 ng/dl) were lower than the normal limits, while the other hormone levels including TSH:1.12 μIU/ml (1.3−16 μIU/ml), free T4: 13.56 pg/ml (9−26 pg/ml), growth hormone (GH): 23.79 ng/ml (normal levels > 20 ng/ml), PRL: 229.25 ng/ml (30−495 ng/ml), cortisol: 12.43 μg/dl (2−11mg/dl) were within the normal ranges (7−9). His urine density was 1000 with polyuria (urinary flow 3.5 ml/kg/hours) and he had hypernatremia. Central diabetes insipidus was diagnosed and desmopressin acetate was given sublingually. Other biochemical and hematological parameters were found normal. The patient was discharged at the end of the third week because there was no further indication for hospitalization. After wards we were informed that he died at home one week after being discharged. The cause of his death was unknown.

## PATIENT 2

**Figure 3:**
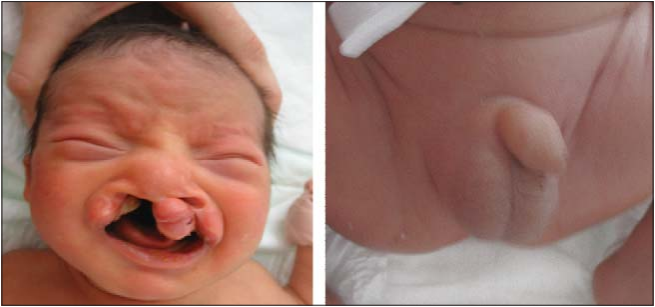
Bilateral cleft lip and plate, hypertelorism, micropenis and cryptorchidism of case 2

**Figure 4:**
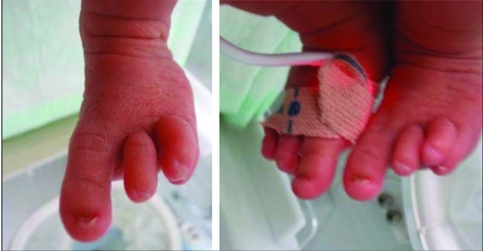
Ectrodactyly deformity of the feet in case 2

A 2800−gram male baby was born at 37−week gestation, by spontaneous vertex delivery. He cried immediately after birth. He was examined when he was 5 days old. His mother’s antenatal history was normal. His mother was 25 years old and his father was 29 years old and they were nonconsaguineous, and the patient was their single child. His family history was normal. He was examined when his weight was 2600 gr (25−50^th^ centile), length 47 cm (25−50^th^ centile), head circumference 32.5 cm (25−50^th^ centile) and chest circumference 30 cm. The baby had midfacial hypoplasia, hypertelorism, and bilateral complete cleft lip and palate deformity. On genital examination micropenis was detected (penile length 2.2 cm (normal: 2.5−4.5 cm)) and bilateral testes were nonpalpable in the scrotum (Figure 3). The skin was dry and hair was thin. The nails of both hands and feet were dystrophic. Ectrodactyly of the feet was observed and both feet had 4 fingers (Figure 4). Ophthalmological examination revealed lacrimal punctal stenosis. Cardiological and renal evaluations were normal. His adenohypophyseal height was measured as 2.5 mm (normal range: 3.5±0.5) (6) on the magnetic resonance imaging. GH (15.43 ng/ml) (normal level >20 ng/ml), FSH (0.57 UI/l) (2.91±4.38 UI/l), LH (0.29 UI/l) (2.31±2.29 UI/l) and total testosterone (12 ng/dl) levels were lower than normal (7, 8, 9). TSH (4.52 μIU/ml), free T4 (15.43 pg/ml), PRL (162.38 ng/ml), cortisol (6.79. mg/dl) were within the normal ranges (8). His biochemical and hematological parameters, and urine analysis were normal. In the follow up, the patient underwent reconstructive operation for the cleft lip and palate when he was 6 months old and for cryptorchidism at the age of 8 months. His growth rate was observed later as insufficient. His height increased 10 cm in the first year. This poor growth rate was associated with insufficient GH levels.

## DISCUSSION

The characteristics of EEC syndrome are ectrodactyly (split hand/foot deformity), ectodermal dysplasia, and cleft lip and/or palate (10). 

The word ectrodactyly means congenital absence of all or part of one or more fingers or toes which results from abnormal development in median rays. It is synonymous with split hand or foot deformity, or lobster claw. Cleft palate with variable lip involvement is seen in 68−100% of the cases (11). Features of ectodermal dysplasia in 77% of the patients include sparse hair, dystrophic nails, hypopigmentation and abnormal dentition (11). Microcephaly and mental retardation have been reported in about 5−10% of the cases (10). Anomalies of lacrimal ducts are common in 59% and epiphora and recurrent infections with keratitis are commonly induced by nasolacrimal duct obstruction. Almost half of the patients have some degree of conduction deafness (4). In addition, urogenital defects including vesicoureteral reflux, kidney anomalies, recurrent urinary tract infections, have been reported (11, 12).

Previously, a family with ectrodactyly and ectodermal dysplasia (hypotrichosis and abnormal dentition) without cleft lip/palate was reported by Wallis et al (13). Anneren et al (14) suggested that low birth weight and polysyndactyly without ectrodactyly may be features of the EEC syndrome. 

The phenotypic variations of this syndrome may be the result of the variable penetrance of specific genes. This syndrome is rare and is usually inherited in an autosomal−dominant manner attributed to mutations in gene encoding p63, a tumor suppressor protein, involving both the ectodermal and mesodermal tissues (15, 16). 

Roelfsema et al (11) determined diagnostic criteria in a retrospective analysis of 230 cases of the EEC syndrome. According to this report, diagnosis of isolated cases of EEC syndrome can be ascertained when two of the three cardinal symptoms are present, while familial cases are diagnosed with only one cardinal symptom in addition to the presence of a first−degree relative with definitive EEC syndrome. They suggested that isolated cases were more severely affected than familial cases. 

Hormonal disorders in EEC syndrome have been defined in few case reports. Van Maldergem et al (17) previously described hypogonadotropic hypogonadism in a boy with EEC syndrome. 

Knudtzon and Aarskog (18) reported EEC syndrome with isolated growth hormone deficiency and absent septum pellucidum in two children. They concluded that developmental hypothalamic defects may lead to isolated growth hormone deficiency.

Hypothalamic and pituitary developmental defects are reported in children who have isolated cleft lip and palate (19, 20).

Gershoni−Baruch et al (21) reported, hypogonadotropic hypogonadism, and a small pituitary gland on MRI in two brothers with EEC syndrome. One of the siblings had GH, TSH and prolactin insufficiency. They suggested that hypothalamo−pituitary insufficiency may be one of the various manifestations of EEC syndrome (21).

Since hypothalamo−pituitary endocrinopathy may be a characteristic feature of midline defects, a decreased secretion of anterior pituitary hormones such as GH, ACTH, TSH, PRL, FSH and LH is expected in these cases. Thus, children with midline defects need not only to be carefully monitored for their growth rate, but also for their pubertal development and bone maturation. Assessment of hormonal status is necessary in children with midline defects to start treatment early.

We reported two boys with EEC syndrome and hypogonadism. Both of them had micropenis and bilateral cryptorchidism. Their gonadotropin levels were lower than the normal range. On cranial MRI small pituitary gland was shown and it was 2.5 mm in height (6). First patient was considered to have diabetes insipidus which responded to desmopressin treatment. The second patient had GH deficiency and hypogonadotropic hypogonadism. In newborns, random serum GH level less than 20 ng/ml may be considered as GH deficiency according to the consensus guidelines for the GH deficiency in childhood and adolescence (9). We considered that these two cases might be sporadic cases since their families were normal. Alternatively, this could be the result of an autosomal or X−linked recessive disorder. Unfortunately we did not perform genetic testing on these patients.

We suggest that one of the various clinical manifestations of EEC syndrome may be hypothalamo−pituitary insufficiency. Therefore, this situation should be kept in mind and should be investigated in cases of EEC syndrome.
